# Association of Genetic Polymorphisms in *TNFRSF11* with the Progression of Genetic Susceptibility to Gastric Cancer

**DOI:** 10.1155/2020/4103264

**Published:** 2020-06-22

**Authors:** Yu Fan, Xuyu Gu, Huiwen Pan, Zhe Dai, Chen Zou, Zhenjun Gao, Heng Zhang

**Affiliations:** ^1^Cancer Institute, The Affiliated People's Hospital of Jiangsu University, Zhenjiang, Jiangsu, China; ^2^Digestive Department, Qingpu Branch Hospital of Affiliated Zhongshan Hospital, Fudan University, Shanghai, China; ^3^Department of General Surgery, Nanjing Lishui District People's Hospital, Zhongda Hospital Lishui Branch, Southeast University, Nanjing, China

## Abstract

**Objective:**

To investigate the relationship between polymorphism of *TNFRSF11* gene *rs9533156* and *rs2277438* and susceptibility to gastric cancer.

**Methods:**

A case-control study was conducted to select 577 cases of primary gastric cancer and 678 cases of normal control. We extracted whole blood genomic DNA and amplified the target gene fragment by PCR. The genotyping and allele were tested through the snapshot method.

**Results:**

In this case-control study, we observed that there was a difference in the genotype distribution of *TNFRSF11* gene *rs9533156* between the case group and the control group. The frequency distribution of TC heterozygous mutation in the case group was higher than that in the control group. The smoking rate in the case group (34.49%) was higher than that in the control group (27.29%), and the difference in frequency distribution between the two groups was statistically significant (*P*=0.006). Our findings suggest that *TNFRSF11 rs9533156* is associated with susceptibility to GC, which is more evident among elderly patients (>62 years), nonsmokers, and patients who do not consume alcohol. The analysis of the relationship between the *TNFSF11* gene *rs9533156* site variant and clinical factors of gastric cancer showed that, compared with the tumor size <2 cm group, patients with tumor size ≥2 cm and whom carrying *rs9533156* site mutations had a higher frequency distribution, and the difference was statistically significant (*P*=0.022). Compared with the nonhyperglycemic group, the frequency distribution of patients with *rs9533156* site mutations in the diabetes group was higher, and the difference was statistically significant (*P* < 0.001).

**Conclusion:**

This study shows that there is a correlation between smoking and the occurrence of gastric cancer. Based on our research, the functional SNP *TNFRSF11* TC genotype may be an indicator of individual susceptibility to GC. The mutation at *rs9533156* may be related to the size of gastric cancer. The mutation rate of *rs9533156* of *TNFSF11* gene is higher in diabetic gastric cancer patients.

## 1. Introduction

Gastric cancer is one of the most common malignant tumors in China. Due to its insidious onset and the current lack of effective early diagnostic molecular markers, patients often found to be advanced, resulting in a 5-year survival rate <25%. Local and distant metastasis of gastric cancer is the main reason for the low survival rate in patients with gastric cancer. Invasion and metastasis of gastric cancer is a complicated process involving multiple factors such as tumor biological behavior, metastasis pathway, and characteristics of metastatic organs. And individual genetic factors play an important role. Single nucleotide polymorphism (SNP) refers to the polymorphism of DNA sequence caused by the variation of a single nucleotide in the genome, which is the most common form of human genetic variation, accounting for more than 90% of all known polymorphisms [1]. Single nucleotide polymorphisms (SNPs) can regulate gene function and expression and has become an effective tool and means of genetic research. *TNFRSF11* (tumor necrosis factor (ligand) superfamily member11), also known as OPGL (osteoprotegerin ligand) and RANKL (receptor activator of NF- kappa B ligand), is a type II transmembrane protein expressed on the surface of bone marrow stromal cells, osteoblasts, T lymphocytes, etc. [2–4]. *TNFRSF11* is expressed in different organs of the human body, such as liver, bone, muscle, intestinal tract, adrenal gland, and other tissues [5]. *TNFRSF11* (RANK) was initially identified and demonstrated to play a role in bone dissolution and lymph node development mainly through the RANK/RANKL/OPG pathway [6].

Recently, the RANK/RANKL/OPG system has been observed to play a key role in tumor cell migration and invasion [7–9]. Studies have shown that *TNFRSF11*(RANK) is expressed in a variety of tumor tissues, such as osteosarcoma [10, 11], chondrosarcoma [12], breast cancer [13], prostate cancer [14, 15], renal cancer [16], oral squamous cell carcinoma [17], lung cancer [18], thyroid cancer [19], and melanoma [20]. However, the expression of *TNFRSF11* in gastric cancer cells is rarely reported, and there is no report on the relationship between *TNFRSF11* gene polymorphism and gastric cancer. Therefore, the present study used a case-control method to compare the genotypes and alleles of *TNFRSF11* gene *rs9533156* and *rs2277438* in gastric cancer patients and healthy controls to analyze the relationship between *TNFRSF11* gene polymorphism and gastric cancer susceptibility. At the same time, combined with the patient's clinical parameters, such as gender, age, smoking history, and drinking history, we can analyze the correlation between them comprehensively thus providing a theoretical basis for early screening and early treatment of gastric cancer.

## 2. Research Object

A total of 577 patients (case group) with gastric cancer admitted to Zhenjiang First People's Hospital from May 2013 to June 2017 were included. The average age of the case group was 61.34 ± 11.097 years, including 394 males and 183 females, all of whom are Chinese, ethnic Han. All cases were clinically and pathologically confirmed to be gastric cancer, and all cases were primary gastric cancer, excluding secondary or recurrent tumors and other malignant tumors. None of the patients underwent radiotherapy or chemotherapy. A total of 678 healthy physical examinees from the physical examination center of Zhenjiang First People's Hospital were selected as the control group. The average age of the people in the control group was 62.31 ± 7.549 years old, including 456 males and 222 females. The people in the control group had no previous genetic history of the tumor. The control group was matched with the case group in terms of gender and age (there was no statistically significant difference in the *t*-test distribution of age and gender between the two groups (*P* > 0.05)), and there was no blood relationship between the two groups.

## 3. Research Method

We followed the methods of Ding et al. [21]. Relevant disease history information of all study subjects was collected by researchers who had all been uniformly trained before. Also, double-entry and logical proofreading were used to ensure the accuracy of the input information. The experimental equipment was provided by the central laboratory of Zhenjiang First People's Hospital, and the main reagents were procured from the Furui Bioengineering Co., Ltd., Shanghai. The whole experiment process was guided and carried out by specialized and licensed laboratory technology operators to reduce any possible errors that might occur throughout the process, improving the accuracy of the results. 2 ml peripheral venous blood of all study subjects from two groups was collected by professional nurses strictly following the principle of sterilization. Then, peripheral blood samples were processed with EDTA anticoagulation and stored at −20°C for later use. After the centrifuge of the plasma, genomic DNA was extracted according to the instructions provided. Before being applied to further experiment, the purity of extracted genomic DNA needed to be determined. Any samples that did not meet the ratio requirements were discarded. The genomic DNA was extracted again until the ratios of OD280/OD230 and OD260/OD280 reached the normal ratio requirements for the experiment. Therefore, it can be ensured that the extracted DNA samples all met the experimental requirements. Primer Premier 5 software was applied in combination with the NCBI database to design upper and lower primers and snapshot extension primers of *TNFRSF11* gene *rs9533156* and *rs2277438*, respectively. The primers were synthesized with technology help from the Furui Bioengineering Co., Ltd., Shanghai. *TNFRSF11-rs9533156* F: 5′-*AACTGTATCATCAGCTTCGTGT*-3′. The content of G + C was 40.91%, and the Tm value was 57.81°C. *TNFRSF11*-*rs9533156* R: 5′-*TGAAGGTGACATTGAGCGAGG*-3′. The content of G + C was 52.38%, and the Tm value was 60.34°C. The product with a length of 190 bp was obtained by PCR amplification. T*NFRSF11*-*rs2277438* F: 5′-*CCTGTGGATGATAGTCAGTTACTCG*-3′. The G + C content is 48.00%, and the Tm value was 60.51%. *TNFRSF11-rs2277438* R: 5′-*AGGAGGAGAAACAGTAAGGACG*-3′. The G + C content was 50.00%, and the Tm value was 59.17%. The length of the product was 201 bp by PCR amplification. The PCR amplification products were purified by ExoI and FastAP for extension reaction, and the genotyping was performed with the ABI3730XL sequencing apparatus after. Allele-specific primer extension was performed with ddNTP labeled with fluorescent dye using the snapshot method for the detection of gene polymorphism. The snapshot method mainly consists of three basic steps: amplification, primer extension, and analysis. Genotyper or GeneMapper software designed for the observed peak color and fragment length range can be used for automated allele analysis. For quality control, 5% of samples were randomly selected for reinspection to ensure the accuracy of the test results.

## 4. Statistical Method

The data were analyzed by using SPSS 20.0 software (SPSS Inc., Chicago, Illinois, USA). The frequency of genotype distribution in the control group and case group was tested using the Hardy-Weinberg genetic balance test. Chi-square test was used to compare the correlation between genotype and allele frequency distribution and NSCL/P and calculate the *X*^2^ value and *P*value. Binary logistic regression analysis was used to calculate the odds ratio (OR) and its 95% confidence interval (CI) to analyze the relative risk of disease caused by genotyping or alleles at polymorphic sites. The statistical tests were all two-sided probability tests; *P* < 0.05 was considered statistically significant.

## 5. Results

The results in Table 1 show the basic information of the *TNFRSF11* gene *rs9533156* and *rs2277438* polymorphism: the genotype frequency distribution of the control group Hardy-Weinberg equilibrium (*P*value for HWE, all *P* > 0.05, Table 1).

The results in Table 2 show the characteristics of the study subjects, including demographics and environmental risk factors. The smoking rate was much higher in the case group as compared with the control group (34.49% versus 27.29%, *P* =0.006). The demographics (age and sex) were well matched (*P*=0.635 and *P*=0.698, respectively). That indicated the occurrence and development of smoking and gastric cancer. The drinking rate was lower in the case group as compared with the control group, but there was no statistically significant difference between the two groups (21.49% versus 23.30%, *P*=0.443).

The results in Table 3 show that the frequency distribution and logistic regression analysis of the *rs2277438* polymorphism of *TNFSF11* gene in gastric cancer and control group showed that the wild-type AA was used as the reference type, and the frequency distribution of AG heterozygous mutant type at *rs2269700* was higher in the control group than that in the case group (43.76% > 41.19%). However, although the difference between the two groups was not statistically significant (*P*=0.310), after logistic regression adjustment analysis based on gender, age, smoking, and drinking, there was still no statistical difference but it was closer to the difference than the results before the regression (*P*=0.263). The frequency distribution of GG homozygous mutants was also not statistically significant (*P*=0.919), and there was no statistical difference after logistic regression adjustment (*P*=0.427). In the dominant model, the frequency distribution of AG + GG mutations was not statistically significant in the case-control group (*P*=0.947), and the difference was not statistically significant after regression adjustment (*P*=0.228). In the recessive model, the frequency distribution was not statistically different (*P*=0.822). According to gender, age, smoking, and drinking, after logistic regression analysis, there was still no statistical difference between the two groups (*P*=0.681).

The results in Table 4 show that the results of the frequency distribution and logistic regression analysis of *TNFRSF11* gene *rs9533156* in gastric cancer and control group showed that the difference between the two groups with the wild-type TT as the reference type was statistically significant (*P*=0.044), and the difference was statistically significant after logistic regression analysis adjusted according to gender, age, smoking, and drinking (*P*=0.039). There was no statistically significant difference in the frequency distribution of CC homozygous mutant (*P*=0.318), and there was no statistically significant difference after logistic regression analysis (*P*=0.294). In the distribution of the dominant model\recessive model, there was no statistical difference (*P*=0.062; 0.056). According to logistic regression analysis, there was still no statistical difference (*P*=0.918; 0.909).

The results in Table 5 show that, compared with the frequency distribution of the A allele in rs2277438, the G allele was lower in the case group than that in the control group (29.84% < 31.50%), but the difference was not statistically significant (*P*=0.372). Also, the frequency distribution of the C allele in rs9533156 was not statistically significant in the case-control group (*P*=0.264).

The results in Table 6 show that, according to stratification results, the polymorphism of *TNFRSF11* gene rs2277438 showed that, with wild-type AA as a reference genotype, there was no statistical significance in the distribution of heterozygous AG and homozygous TT. In the dominant model and recessive model, the difference in gene distribution was not statistically significant. And no significant differences were found in wild-type TC, homozygous TT, dominant models, and recessive models after stratified analysis according to gender, age, smoking, and drinking factors.

Table 7 summarizes the results of the association between *TNFRSF11* gene rs9533156 polymorphism and GC risk in the stratified analysis. In the elderly (≥62 years old) group, the frequency distribution of TC heterozygous mutations was statistically different between the two groups (*P*=0.002), and the risk of gastric cancer in the TC group was 1.83 times higher than that of the TT type. TC mutant mutation may be a risk factor for gastric cancer. In the c-dominated dominance model, the frequency distribution of TC + CC was statistically different between the two groups (*P*=0.004), and the risk of developing gastric cancer in the TC + CC group was 1.70 times higher than that in the TT group. The c-dominated TC + CC group may be a risk factor. In the nonsmoker group, the frequency distribution of TC heterozygous mutations was statistically different between the two groups (*P*=0.017), and the risk of gastric cancer in the TC group was 1.49 times higher than that of the TT type. In the c-dominated dominance model, the frequency distribution of TC + CC was statistically different between the two groups (*P*=0.016), and the risk of developing gastric cancer in the TC + CC group was 1.46 times higher than that in the TT group. In the group of nondrinkers, the frequency distribution of TC heterozygous mutations was statistically different between the two groups (*P*=0.043), and the risk of gastric cancer in the TC group was 1.37 times higher than that of the TT type. In the c-dominated dominance model, the frequency distribution of TC + CC was statistically different between the two groups (*P*=0.037), and the risk of developing gastric cancer in the TC + CC group was 1.36 times higher than that in the TT group.

The results in Table 8 show that *TNFSF11* gene *rs9533156* site variants and gastric cancer clinical factor analysis results show that, compared with the tumor size <2 cm group, patients with tumor size ≥2 cm and carrying *rs9533156* site mutations had a higher frequency distribution, and the difference was statistically significant (*P*=0.022). This suggests that *rs9533156* mutation may be related to the size of gastric cancer. Compared with the nonhyperglycemic group, the frequency distribution of patients with hyperglycemia (diabetes group) who also carry the *rs9533156* mutation is higher, and the difference was statistically significant (*P* < 0.001). This suggests that hyperglycemia and *rs9533156* site mutation are related risk factors for gastric cancer. There was no significant correlation between *rs9533156* and the location, differentiation, and pathological type of gastric cancer. However, there is no statistical association between the *rs2277438* locus variant and the clinical factors of gastric cancer.

## 6. Discussion

Gastric cancer is a multifactorial disease including diet, genetic factors, environmental factors, immune factors, infections, and inflammation. SNP can modulate gene function and/or expression, and SNP-association studies may provide important insights into the pathogenesis of gastric cancer. At present, studies on the *TNFRSF11* gene mainly focus on the effect and mechanism of RANK/RANKL/OPG bone regulatory pathway on osteoporosis/fracture healing, while there are few studies on the polymorphism of TNFRSF11 gene. Castilhos et al. [22] found that the longer roots of upper central incisors and rapid maxillary expansion, as well as allele A of the rs3102724 polymorphism of the OPG gene and EARR, were associated with EARR in Brazil's white people. Mrozikiewicz-Rakowska et al. [23] found that the following variants *TNFRSF11 B* (rs2073618, rs2073617, rs1872426, rs1032128, rs7464496, rs11573829, and rs1485286), COLEC10 (rs6993813 and rs3134069), and *TNFSF11* (rs9533156) present differences in allele frequencies in diabetic foot patients and show correlation with gender, diabetes type, and diabetic foot etiology. Casas-Avila et al. [24] found that carrying the GG genotype of rs12585014 entails a higher risk of having menarche later (>13 years), which could involve a greater risk of fractures. The rs3018362 and rs12585014 do not seem to be associated with hip osteoporosis or hip fracture in Mexican women. Sassi et al. [25] also found the association of the-643-c > T polymorphism with BMD variation and osteoporosis risk in postmenopausal Tunisian women. Shaker and Senousy [26] found that there are interactions between RANKL-rs9533156 and OPG-rs2073618. A stronger combined effect of SNPs in RANKL and OPG genes via gene-gene interaction may help predict breast cancer risk and prognosis. Lucas Corso et al. [27] found that the polymorphism rs2073618 of OPG is a possible marker that is associated with the risk of the manifestation of TMJ ankylosis. Our team [28] also found that functional SNP RNK rs 1805034 T > C may be an indicator of susceptibility to esophageal squamous cell carcinoma (ESCC). However, the correlation between *TNFRSF11* gene polymorphism and gastric cancer susceptibility has not been reported.

In this study, we selected 577 gastric cancer patients and 678 healthy volunteers to explore the relationship between *TNFRSF11* gene rs9533156 and rs2277438 loci and gastric cancer susceptibility in Chinese Han population. This study found that smoking was correlated with the occurrence and development of gastric cancer and was an independent risk factor for gastric cancer. However, there was no significant difference in the distribution of alcohol consumption between the two groups. In this hospital-based case-control study of GC, we investigated the association of *TNFRSF11* gene rs9533156 and rs2277438 polymorphism with the risk of GC. We found that the *TNFRSF11* gene rs9533156 TC SNP was significantly associated with an increased risk of GC. It is worth noting that elderly patients (>62 years old) carrying the rs9533156 TC genotype of *TNFSF11* gene, nonsmokers, and patients who do not drink alcohol have an increased risk of GC after stratification analyses.

In a further study on the relationship between *TNFSF11* gene locus and clinical parameters of gastric cancer, we found that rs9533156 mutation may be related to the tumor size of gastric cancer. Compared with the group with tumor size <2 cm, the patients with tumor size ≥2 cm carrying rs9533156 mutation at the same time had higher frequency distribution, and the difference was statistically significant. This also confirms our previous research conclusion that rs9533156 TC genotype may be a risk factor for gastric cancer. Compared with the nonhyperglycemia group, the frequency distribution of the patients with hyperglycemia (diabetes group) carrying rs9533156 mutation at the same time was higher, and the difference was statistically significant. There was no significant correlation between rs9533156 mutation and tumor location, and differentiation degree and pathological type. There was no significant correlation between rs2277438 and clinical factors of gastric cancer.

To our knowledge, this study is the first to show a significant correlation between *TNFRSF11* rs9533156 TC genotype and gastric cancer susceptibility. Although the association appears to be statistically significant, the findings need to be replicated in large independent samples to further confirm the role of *TNFRSF11* in genetic susceptibility to gastric cancer. Several limitations of this study need to be addressed. First, the patients and controls were recruited from hospitals and may, therefore, not be representative of the general population. Second, the SNPs studied may not provide a comprehensive picture of the *TNFRSF11* genetic variability. Third, *Helicobacter pylori* is generally regarded as the main cause of peptic ulcer disease and gastric adenocarcinoma. However, due to insufficient patient clinical information, we did not investigate *Helicobacter pylori* infection. Thus, the power of our analyses was restricted.

However, our study found that the mutation rate of rs9533156 in the *TNFSF11* gene of diabetic gastric cancer patients is higher, suggesting that diabetes may be related to gastric cancer, and diabetic patients have a higher risk of gastric cancer. Previous studies have also found that patients with diabetes have an increased risk of cancer [29]. However, there are not many reports on the relationship between diabetes and gastric cancer. Whether type 2 diabetes increases the risk of gastric cancer remains controversial. Miao et al. [30] reported the data of 8559861 participants in a meta-analysis of 22 cohort studies and found that diabetes was an incentive to increase the risk of gastric cancer in men. However, other studies have reported conflicting results of women's cancer risk. Inoue et al. [31] conducted a group analysis in the 2011 meta-analysis and found that, compared with men with diabetes, women with diabetes had an 18% increased risk of stomach cancer.

In 2012, American and European scholars believed that type 2 diabetes had a significant positive effect on gastric cancer [32, 33]. However, Khan et al. previously believed that type 2 diabetes can effectively reduce the incidence of gastric cancer [34]. Also, in a 2015 study by Xu et al., there was no significant correlation between the risk of gastric cancer and diabetes among men and women [35]. Increased levels of insulin and insulin-like growth factor 1 receptor in diabetic patients can promote cell division, inhibit apoptosis, and promote proliferation and differentiation [36]. Hyperglycemia can induce cell damage, and glycosylated substance can promote the production of oxygen free radicals and DNA damage [37]. Besides, glucose may act as a source of energy to promote the development of gastric cancer, especially for the types with rapid growth and a high degree of malignancy. Also, the immune function of patients with diabetes is reduced, the internal environment is disordered, and chronic inflammation and the use of drugs such as sulfonylurea, metformin, and insulin also increase the risk of gastric cancer. Of course, the mechanism that causes this correlation is not yet clear, and it may be related to multiple channels.

In conclusion, our study provides evidence that genotype mutation of *TNFSF11* gene rs9533156 TC may be associated with GC risk. To confirm our findings, tissue-specific biology and large population replication studies are needed.

## Figures and Tables

**Table 1:** Primary information of *TNFRSF11* gene *rs9533156*, *rs2277438* polymorphisms.

Genotyped SNPs	Gene	Chr pos (NCBI build 38)	Category	MAF^a^ for Chinese in database	MAF in our controls (*n* = 678)	*P* value for HWE^b^ test in our controls	Genotyping method	Genotyping value (%)
*rs9533156*	13	*TNFRSF11*	Intron_variant	0.439	0.315	0.719	Snapshot	98.64
*rs2277438*	13	*TNFRSF11*	Intron_variant	0.300	0.463	0.374	Snapshot	95.94

^a^Minor allele frequency and ^b^Hardy–Weinberg equilibrium.

**Table 2:** Distribution of selected demographic variables and risk factors in gastric cancer cases and controls.

		Overall cases (*n* = 577)	Overall controls (*n* = 678)	*P*
		*n* (%)	*n* (%)	
Age (years)		61.34 ± 11.097	62.31 ± 7.549	0.065

Age (years)				
<62		268 (46.45)	324 (47.79)	
≥62		309 (53.55)	354 (52.21)	0.635

Sex				
Male		394 (68.28)	456 (67.26)	
Female		183 (31.72)	222 (32.74)	0.698

Smoking status				
Never		378 (65.51)	493 (72.71)	
Ever		199 (34.49)	185 (27.29)	**0.006**

Alcohol use				
Never		453 (78.51)	520 (76.70)	
Ever		124 (21.49)	158 (23.30)	0.443

**Table 3:** *TNFRSF11* gene *rs2277438* polymorphism in GC cases and controls and logistic regression analysis.

Genotype	GC cases (*n* = 577)		Controls (*n* = 678)		Crude OR (95% CI)	*P*	Adjusted OR (95% CI)	*P*
	*n*	(%)	*n*	(%)				
*rs2277438*								
AA	284	49.56	310	46.62	1.00		1.00	
AG	236	41.19	291	43.76	1.13 (0.89–1.43)	0.310	0.87 (0.69–1.11)	0.263
GG	53	9.25	64	9.62	1.02 (0.68–1.53)	0.919	0.92 (0.75–1.13)	0.427
AG + GG	289	50.44	355	53.38	1.00 (0.80–1.27)	0.947	0.93 (0.83–1.04)	0.228
GG	53	9.25	64	9.62	0.96 (0.65–1.40)	0.822	0.96 (0.79–1.17)	0.681
AA + AG	520	90.75	601	90.38	1.001		1.00	

**Table 4:** *TNFRSF11* gene *rs9533156* polymorphism in GC cases and controls and logistic regression analysis.

Genotype	GC cases (*n* = 577)		Controls (*n* = 678)		Crude OR (95% CI)	*P*	Adjusted OR (95% CI)	*P*
	*n*	(%)	*n*	(%)				
*rs9533156*								
TT	139	24.91	192	29.72	1.00		1.00	
TC	296	53.05	310	47.99	1.32 (1.00–1.73)	**0.044**	1.33 (1.02–1.75)	**0.039**
CC	123	22.04	144	22.29	1.18 (0.85–1.63)	0.318	1.09 (0.93–1.29)	0.294
TC + CC	419	75.09	454	70.28	1.28 (0.99–1.65)	0.062	1.13 (0.99–1.29)	0.056
CC	123	22.04	144	22.29	0.99 (0.75–1.30)	0.918	0.99 (0.86–1.14)	0.909
TT + TC	435	77.96	502	77.71	1.00		1.00	

**Table 5:** Analysis of *rs9533156* and *rs2277438* alleles between cases and controls.

Locus	Variable	Case	Control	*P*	OR (95% CI)
*rs2277438*	A allele	804 (70.16)	911 (68.50)		
	G allele	342 (29.84)	419 (31.50)	0.372	0.93 (0.78–1.09)

*rs9533156*	T Allele	574 (51.43)	694 (53.72)		
	C Allele	542 (48.57)	598 (46.28)	0.264	1.09 (0.93–1.29)

**Table 6:** Stratified analyses between *rs2277438* polymorphism and risk by sex, age, smoking status, and alcohol consumption.

Variable	(Case/control)			Adjusted OR (95% CI); *P*			(AG + GG) VSAA	GGVS (AA + AG)
	AA	AG	GG	AA	AG	GG		
Sex								
Male	198/201	157/198	36/45	1.00	0.81 (0.60–1.07); *P*: 0.138	0.81 (0.50–1.31); *P*: 0.395	0.81 (0.61–1.06); *P*: 0.121	0.90 (0.57–1.43); *P*: 0.651
Female	86/109	79/93	17/19	1.00	1.08 (0.71–1.63); *P*: 0.725	1.13 (0.56–2.31); *P*: 0.729	1.09 (0.73–1.61); *P*: 0.679	1.09 (0.55–2.18); *P*: 0.795

Age								
<62	129/146	108/138	29/31	1.00	0.89 (0.63–1.25); *P*: 0.491	1.06 (0.61–1.85); *P*: 0.841	0.92 (0.66–1.27); *P*: 0.606	1.12 (0.66–1.91); *P*: 0.675
≥62	155/164	128/153	24/33	1.00	0.89 (0.64–1.22); *P*: 0.457	0.77 (0.44–1.36); *P*: 0.367	0.87 (0.64–1.18); *P*: 0.353	0.82 (0.47–1.41); *P*: 0.464

Smoking status								
Never	189/230	154/207	33/49	1.00	0.91 (0.68–1.20); *P*: 0.492	0.82 (0.51–1.33); *P*: 0.417	0.89 (0.68–1.16); *P*: 0.392	0.86 (0.50–1.36); *P*: 0.517
Ever	95/80	82/84	20/15	1.00	0.82 (0.54–1.26); *P*: 0.367	1.12 (0.54–2.24); *P*: 0.757	0.87 (0.58–1.30); *P*: 0.493	1.24 (0.61–2.49); *P*: 0.555

Alcohol consumption								
Never	223/237	182/224	46/52	1.00	0.86 (0.66–1.13); *P*: 0.283	0.94 (0.61–1.46); *P*: 0.782	0.88 (0.68–1.31); *P*: 0.314	1.01 (0.66–1.53); *P*: 0.974
Ever	61/73	54/67	7/12	1.00	0.97 (0.59–1.58); *P*: 0.886	0.70 (0.26–1.88); *P*: 0.476	0.94 (0.58–1.51); *P*: 0.796	0.71 (0.27–1.86); *P*: 0.485

**Table 7:** Stratified analyses between *rs9533156* polymorphism and risk by sex, age, smoking status, and alcohol consumption.

Variable	(Case/control)			Adjusted OR (95% CI); *P*			(TC + CC) VSTT	CCVS (TT + TC)
	TT	TC	CC	TT	TC	CC		
Sex								
Male	91/129	206/210	84/95	1.00	1.39 (1.00–1.93); *P*: 0.050	1.25 (0.84–1.87); *P*: 0.265	0.93 (0.67–1.30); *P*: 0.671	1.01 (0.72–1.41); *P*: 0.957
Female	48/63	90/100	39/49	1.00	1.18 (0.74–1.89); *P*: 0.488	1.05 (0.59–1.84); *P*: 0.879	1.14 (0.73–1.77); *P*: 0.572	0.94 (0.58–1.52); *P*: 0.800

Age								
<62	79/89	118/143	61/69	1.00	0.93 (0.63–1.37); *P*: 0.713	0.99 (0.63–1.58); *P*: 0.986	0.95 (0.66–1.37); *P*: 0.787	1.00 (0.68–1.48); *P*: 0.997
≥62	60/103	178/167	62/75	1.00	1.83 (1.25–2.68); *P*: **0.002**	1.42 (0.89–2.26); *P*: 0.138	1.70 (1.18–2.45); *P*: **0.004**	0.94 (0.64–1.37); *P*: 0.740

Smoking status								
Never	87/146	201/227	80/96	1.00	1.49 (1.07–2.06); *P*: **0.017**	1.40 (0.94–2.08); *P*:0.098	1.46 (1.07–1.99); *P*: **0.016**	1.08 (0.77–1.51); *P*: 0.654
Ever	52/46	95/83	43/48	1.00	1.01 (0.62–1.66); *P*: 0.961	0.80 (0.45–1.40); *P*: 0.425	0.93 (0.59–1.48); *P*: 0.765	0.79 (0.49–1.26); *P*: 0.320

Alcohol consumption								
Never	109/153	235/241	96/101	1.00	1.37 (1.01–1.86); *P*: **0.043**	1.33 (0.92–1.94); *P*: 0.128	1.36 (1.02–1.81); *P*: **0.037**	1.09 (0.80–1.50); *P*: 0.584
Ever	30/39	61/69	27/43	1.00	1.15 (0.64–2.07); *P*: 0.642	0.82 (0.42–1.61); *P*: 0.556	1.02 (0.59–1.77); *P*: 0.940	0.75 (0.43–1.30); *P*: 0.299

**Table 8:** Clinic factors of rs9533156 polymorphism on gastric cancer development.

Group	TC + CC	TT	OR	95% CI	*P* value
Tumor size					
≥2 cm	360 (85.91)	125 (80.13)			
<2 cm	59 (14.09)	31 (19.87)	1.751	1.078–2.845	**0.022**

Tumor location					
Fundus	6 (1.70)	2 (1.85)			
Antrum	256 (72.52)	82 (75.93)	0.961	0.190–4.853	0.962
Body of the stomach	91 (25.78)	24 (22.22)	0.791	0.150–4.171	0.676

Tumor differentiation					
Well	12 (3.47)	2 (1.71)			
Moderate	174 (50.29)	64 (54.70)	2.207	0.481–10.132	0.368
Poor	160 (46.24)	51 (43.59)	1.913	0.414–8.830	0.528

Pathological pattern					
Signet-ring cell carcinoma	17 (4.20)	6 (4.55)			
Both	5 (1.23)	4 (3.03)	0.178	0.183–0.991	0.407
Adenocarcinoma	383 (94.57)	122 (92.42)	0.903	0.348–2.340	0.833

Blood glucose					
Hyperglycemia	114 (27.21)	16 (11.51)			
Nonhyperglycemia	305 (72.79)	123 (88.49)	2.870	1.635–5.048	**<0.001**

## Data Availability

The data used to support the findings of this study are available from the corresponding author upon request.
